# Evolutionary history of bovine endogenous retroviruses in the Bovidae family

**DOI:** 10.1186/1471-2148-13-256

**Published:** 2013-11-20

**Authors:** Koldo Garcia-Etxebarria, Begoña M Jugo

**Affiliations:** 1 Genetika, Antropologia Fisikoa eta Animalien Fisiologia Saila. Zientzia eta Teknologia Fakultatea, Euskal Herriko Unibertsitatea (UPV/EHU), 644 Postakutxa, E-48080 Bilbao, Spain

## Abstract

**Background:**

Endogenous retroviruses (ERVs) are genomic elements of retroviral origin that are present in the genomes of almost all vertebrates. In cattle, more than 13,000 elements related to ERVs have been detected, and based on the *pol* gene, 24 families or groups of bovine ERVs have been described. However, information about ERVs in other bovids and the presence of families of related bovine ERVs in different species of the Bovidae family is scarce.

**Results:**

The 24 families of bovine ERVs previously detected in cattle (*Bos taurus*) were also detected in zebus (*Bos indicus*) and yaks (*Bos grunniens*). In addition, six new families, named BoERV25 to BoERV30, were detected in the three *Bos* species. Five more ruminant species were screened for related ERVs: 26 families were detected in these species, but four families (BoERV24, BoERV26, BoERV28 and BoERV29) were specific to cattle, zebus, yaks and buffalo. An analysis of the homology of the ERVs of cattle, zebus and yaks revealed that the level of LTR divergence was similar between ERVs from cattle and zebus but was less similar between with ERVs from cattle and yaks. In addition, purifying selection was detected in the genes and retroviral regions of clusters of ERVs of cattle, zebus and yaks.

**Conclusions:**

In this work, the 24 ERV families previously identified in cattle were also found in two other species in the *Bos* genus. In addition, six new bovine ERV families were detected. Based on LTR divergence, the most recently inserted families are from Class II. The divergence of the LTR, used as an indirect estimate of the ERV insertion time, seemed to be influenced by the differences in genome evolution since the divergence of the species. In addition, purifying selection could be acting on clusters of ERVs from different species.

## Background

Endogenous retroviruses (ERVs) are genomic elements present in the genomes of almost all vertebrates. They evolved from exogenous retroviruses that were incorporated into the genome and became part of the host genome [1].

In the Bovidae family, ERVs have been analyzed in most detail in domestic sheep (*Ovis aries*) and cattle (*Bos taurus*). The former are a good model of the coevolution of retroviruses and the host genome due to the interaction between the JSRV virus, its endogenous counterpart and the host [2]; and the latter have been studied in depth to detect ERVs [3,4] because the cattle genome was the first to be sequenced among ruminants [5]. In total, more than 13,000 retroviral elements have been detected in cattle. Some of them are classified into 24 bovine endogenous retrovirus (BoERV) families, and because enJSRV has been detected in some breeds but not in others, variability in endogenous retroviruses among breeds has been proposed [4,6].

However, the distribution of related BoERVs in other closely related species remains unknown, although the presence of some ERVs in yaks and goats has been detected by Southern blotting [7]. Fortunately, more and more data have been generated for different species in the Bovidae family. The genomes of zebus (*Bos indicus*) [8], yaks (*Bos grunniens*) [9], water buffalo (*Bubalus bubalis*) [10] and goats (*Capra hircus*) [11] have been recently released, and the genomes of domestic sheep, bighorn sheep (*Ovis canadensis*) and Dall sheep (*Ovis dalli*) are currently being sequenced [12].

The study of related BoERVs harbored by different species could aid in the study of specific evolutionary processes. For example, it may be possible to measure the utility of the use of LTR divergence to estimate the integration time of ERVs because we could detect the ERVs of different species which are inherited from the common ancestor of the three *Bos* species. LTR divergence has been used for this purpose [4,13,14], although there are some concerns about its utility [15]. In addition, the selective pressure affecting different genes of closely related BoERVs could be analyzed because purifying selection has been detected in different ERVs, especially in the *pol* gene [1,16-18].

Thus, using different computational approaches, we analyzed (1) the genome-wide presence of known and new ERVs in cattle, zebus and yaks and (2) the presence of known BoERV families in these species and five other species, namely water buffalo, domestic sheep, bighorn sheep, Dall sheep and goats.

## Results

### Detection and comparison of endogenous retroviruses in cattle, zebus and yaks

The cattle genome was previously analyzed to detect endogenous retroviruses using three different computational approaches [4]. However, to make the results comparable with those for two other species, zebus and yaks, this genome was reanalyzed combining LTRharvest [19], to find elements with long terminal repeats (LTR), and LTRdigest [20], to annotate the elements found. These programs were selected because of their efficiency in time and resources and the easiness to parse the great amount of data generated.

LTRharvest identified 87,340 elements with LTRs in cattle, 55,430 in zebus and 43,624 in yaks (Table 1). Their position in chromosomes or contigs are showed in Additional files 1, 2 and 3. Because some of these elements could represent incomplete proviruses, LTRdigest was used to annotate retroviral features, especially retroviral genes or regions of retroviral genes, that is, *gag*, *pol* or *env* genes or one of the regions of these genes (e. g. reverse transciptase and integrase). In total, 1532 elements from cattle, 771 elements from zebus and 519 from yaks had at least one retroviral gene or region of retroviral genes (Table 1). These elements are more likely to be ERVs because they each contain an LTR and at least one retroviral gene or region of retroviral genes. In the case of cattle, among the 1532 detected elements, 574 were detected in our previous work [4]. In the three species, regions from the *pol* gene (integrase, RNaseH and reverse transcriptase) were the most frequently detected retroviral regions, and the *env* gene was the least frequently detected (Table 1). In addition, the most common structures in the detected ERVs were LTR-*pol*-LTR, numbering 548 (35.77% of elements with retroviral genes or regions of retroviral genes) in cattle, 317 (41.11%) in zebus and 213 (41.04%) in yaks. The complete structure was detected in 251 elements (16.38%) in cattle, 113 (14.66%) in zebus and 75 (14.45%) in yaks.

**Table 1:** **Number of ERVs detected *de novo *** **by LTRHarvest and LTRDigest in each *Bos *** **species and their features**

	***B. taurus***	***B. indicus***	***B. grunniens***
Elements detected	87340	55430	43624
Elements with PPT	22002	14153	10285
Elements with PBS	5154	3279	2963
Elements with retroviral genes or regions of retroviral genes.	1532	771	519
- With *gag*	675	316	199
- With protease	701	338	186
- With *pol*	1324	685	440
- With RT	834	417	240
- With IN	927	475	315
- With RNaseH	816	429	228
- With *env*	537	246	172
- With other	348	132	81
LTR-*gag*-*pol*-*env*-LTR	251	113	75
LTR-*gag*-*pol*-LTR	334	160	91
LTR-*gag*-*env*-LTR	13	8	9
LTR-*pol*-*env*-LTR	191	95	61
LTR-*gag*-LTR	77	35	24
LTR-*pol*-LTR	548	317	213
LTR-*env*-LTR	82	30	27

PPT, polypurine tract; PBS, protein binding site; RT, reverse transcriptase; IN, integrase.

**Figure 1:**
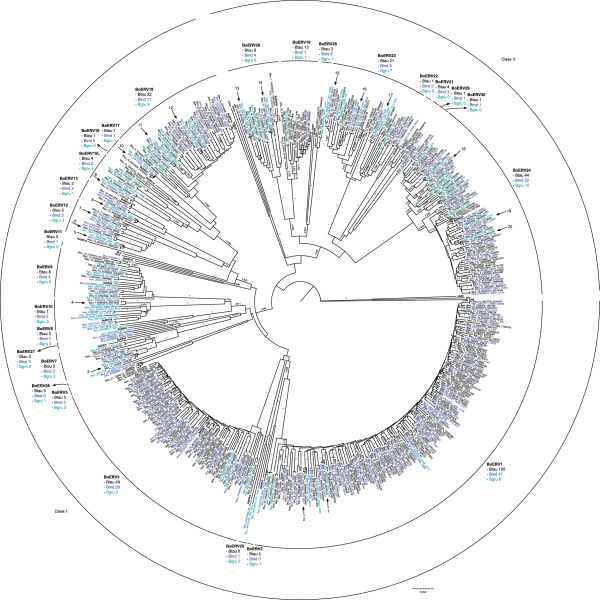
**Phylogenetic tree of ERVs detected in *Bos *genera.** Neighbor-Joining tree (p-distance, pairwaise deletion and 1000 bootstrap) of 300 ERVs from cattle (named as Btau and in black), 156 from zebu (named as Bind and in dark blue), 70 from yak (named as Bgru and in light blue), 24 of bovine ERV families (in bold) and 11 “markers” (in bold). ZAM element from *Drosophila* was used as root. In the case of ERVs from cattle the chromosome and position are showed; in the case of ERVs from zebu and yak the gi number of the contig and position are showed. Clusters of ERVs from *Bos* species are marked with an arrow and labeled with a number.

The elements detected by LTRharvest were better characterized. First, the elements were searched for a reverse transcriptase region using BLAST. A total of 300 ERV sequences from cattle, 156 from zebus and 70 from yaks were used to detect the presence of previously described families of BoERVs (Figure 1). Eighteen out of the 24 families were detected in cattle, 18 were detected in zebus and 17 were detected in yaks. In addition, there were six groups of sequences that did not cluster with previously described families, and these sequences were considered new families and were named BoERV25 to BoERV30. There were also three previously defined families (BoERV4, BoERV6 and BoERV14) that were not detected in this analysis. Last, there was a cluster of sequences that were weakly related to BoERV15 that was named BoERV15-Like (BoERV15L) (Figure 1). All detected families belonged to the Class I (21 families) and Class II (9 families) ERV classes. No Class III family members were detected (Figure 1).

### Presence of bovine ERVs in other species of the Bovidae family

**Figure 2:**
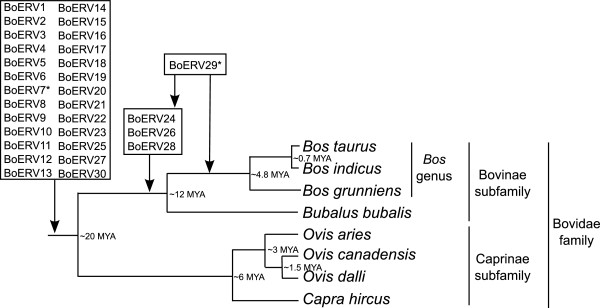
**Presence of families of BoERVs in ruminants.** Presence of families of BoERVs in common ancestor of Bovidae family, Bovinae subfamily and *Bos* genus. Split times are based on [21-23]; MYA, million years ago. * BoERV7 was not detected in *Bubalus bubalis*; reads mapped in BoERV29 in *Ovis canadensis* and *Ovis dalli* but not in *Ovis aries* and *Capra hircus*.

To detect the presence of BoERV families in different species belonging to the Bovidae family, a search for the 30 BoERV families was performed in water buffalo, domestic sheep and goats using BLAST. Due to the lack of assembly of the genomes of buffalo, bighorn sheep and Dall sheep, the MUMmer program was used to map the available reads of these species to the sequences of the *pol* genes of the 30 BoERV families. Twenty-six of the BoERV families were detected in all the species analyzed, including the 3 families that were not detected using LTRharvest and LTRdigest (BoERV4, BoERV6 and BoERV14), and thus, these families of ERVs could be present in all members of the Bovidae family. The analysis of the species different from those analyzed in this work could confirm this point. Four families (BoERV24, BoERV26, BoERV28 and BoERV29) were not detected in sheep or goats, so they seem to be specific to the Bovinae subfamily (Figure 2). Three of these families specific to the Bovinae subfamily were Class II ERVs and one was a Class I ERV. Thus, the majority of the BoERV families were inserted into the genome of the common ancestor of the Bovidae family approximately 20 million years ago (MYA) or earlier. Later, insertions of ancestors of four families could occur in the common ancestor of the Bovinae subfamily, between 12MYA and 20MYA (Figure 2). Strikingly, the BoERV7 family was detected in all species except water buffalo (Figure 2, Table 2). Regarding the insertion time based on LTR divergence, BoERV7 family entered *Bos* species between 34–19 MYA, that is, previous to the divergence of Bovidae family (around 20MYA). Thus, the lack of detection of the BoERV7 family in buffalo could be due to the loss of this family in this species. In the case of BoERV29 family, it was detected in all species except sheep and goat (Figure 2, Table 2). Regarding its insertion, BoERV29 family entered *Bos* species between 29-12MYA, a span of time covering the split of Caprinae and Bovinae subfamilies. Since the majority of reads mapped in a short segment of the query sequence, the high number of reads mapped to BoERV29 in buffalo, bighorn sheep and Dall sheep could be a side-effect of detecting another repetitive element such us BovB LINE. Thus, more species of the Bovidae family should be analysed to clarify the insertion and evolutionary pathway of these families.

**Table 2:** Presence of bovine ERV families in 8 species of ruminants

	**Number of matches in BLAST searches**					**Number of mapped reads**		
**Family**	**Btau**	**Bind**	**Bgru**	**Oari**	**Chir**	**Bbub**	**Ocan**	**Odal**
Previously known								
Class I								
BoERV1	209	139	91	56	84	2363	4419	3263
BoERV2	3	2	3	4	5	8	74	50
BoERV3	187	145	103	101	93	1524	5285	3383
BoERV4	1	1	1	1	1	946	2907	2006
BoERV5	1	1	1	2	3	18	8	11
BoERV6	1	1	1	1	1	33	43	22
BoERV7	1	1	1	1	1	ND	41	41
BoERV8	11	10	12	13	9	35	52	34
BoERV9	23	15	29	28	35	45	249	180
BoERV10	18	12	22	14	13	42	130	59
BoERV11	1	1	1	1	1	29	76	76
BoERV12	1	1	1	1	1	11	4	6
BoERV13	9	9	7	7	7	38	105	59
BoERV14	1	1	1	1	1	13	67	30
BoERV15	1	1	1	1	1	139072	796954	527908
BoERV16	5	8	5	8	9	20	91	63
BoERV17	3	1	1	1	1	15	55	41
BoERV18	47	42	40	43	44	236	2044	1180
Class II								
BoERV19	22	14	17	18	21	485	29	13
BoERV20	20	11	21	17	15	40	80	46
BoERV21	6	2	5	6	5	51	200	151
BoERV22	8	3	7	6	4	36	117	71
BoERV23	40	27	33	25	24	106	538	512
BoERV24	73	56	46	ND	ND	174	ND	ND
Newly detected in this work								
Class I								
BoERV25	4	4	3	3	4	18	65	52
BoERV26	1	1	1	ND	ND	12	ND	ND
BoERV27	6	5	7	8	4	11	41	31
Class II								
BoERV28	7	3	6	ND	ND	101	ND	ND
BoERV29	1	1	1	ND	ND	24991	244188	160149
BoERV30	1	1	1	1	1	9	74	75

ND, no detected.

Btau, cattle; Bind, zebu; Bgru, yak; Oari, domestic sheep; Chir, goat; Bbub, water buffalo; Ocan, bighorn sheep; Odal Dal sheep. In Blast searches, number of matches; in mapping, number of reads mapped.

The number of detected copies of each BoERV family was variable (Figure 1, Table 2). On the whole, the most abundant families, due to the high number of matches or mapped reads, were BoERV1 (the most abundant in cattle), BoERV3 (0.89 times less than BoERV1 in cattle), BoERV18 (0.22 times less than BoERV1 in cattle), BoERV23 (0.11 times less than BoERV1 in cattle) and BoERV24 (0.34 times less than BoERV1 in cattle), while most of the families (e. g. BoERV4, BoERV5 and BoERV6) have one detectable copy (0.0047 times less than BoERV1 in cattle). BoERV1 and BoERV3 were especially abundant in cattle and zebus, as described previously for cattle [4]. In addition, large numbers of reads in the sequencing data for water buffalo, bighorn sheep and Dall sheep mapped to BoERV15 and BoERV29 (Table 2).

### Clusters of ERVs from different *Bos* species

The phylogenetic analysis based on the ERVs detected in *Bos* species (Figure 1) showed that, frequently, ERVs from different species clustered together significantly; these ERVs are likely the same provirus in different species. In total, there were 20 clusters of ERVs (Figure 1). Based on their LTRs and retroviral genes, the integration times and selective pressures acting on these ERVs were analyzed.

Two methods have been applied to estimate the integration time of clustered ERVs: 1) the method proposed by Martins and Villesen [24], which is based on the divergence of the LTRs and the distances based on phylogenetic trees, and 2) the widely used method based on the divergence of LTRs and fixed substitution rates (Table 3). Four clusters of ERVs (the ones labeled 6, 12, 13 and 18 in Figure 1) were not analyzed further because the phylogenetic tree of their LTRs was not consistent with the relationships among the species (data not shown). The estimates of the integration time determined using both methods were similar for 6 clusters of ERVs and different for 10 clusters of ERVs (Table 3). Thus, the use of fixed rate or estimated rate implied a critical difference.

**Table 3:** Estimation of insertion time of clusters of ERVs

**Cluster of ERVs**	**Family divergence**	**Phylogeny based subs. rate**			**Fixed subs. rate**		
		**LTR div / (dist 3′ + dist 5′)**			**LTR div × 2.3x10**^**-6 **^**- LTR div × 5x10**^**-6**^		
		**Btau**	**Bind**	**Bgru**	**Btau**	**Bind**	**Bgru**
1	126-0	19.36	20.47	14.73	30.25-13.92	31.98-14.71	23.01-10.58
2	126-0	26.05	26.89	27.78	24.07-11.07	24.84-11.43	25.67-11.81
3	19-1	4.66	7.28	8.50	19.02-8.75	29.71-13.66	34.70-15.96
4	38-18	25.60	40.34	27.31	20.64-9.49	32.52-14.96	22.01-10.13
5	64-19	98.94	98.94	89.36	35.85-16.49	35.85-16.49	32-38-14.89
6		NA	NA	NA	NA	NA	NA
7	64-19	25.99	25.99	25.29	34.48-15.86	34.48-15.86	33.56-15.44
8	-	60.99	56.30	88.23	21.81-10.04	20.14-9.27	31.57-14.52
9	35-16	36.64	36.64	36.81	32.36-14.88	32.36-14.88	32.51-14.95
10	-	45.55	51.95	45.71	34.04-15.66	38.82-17.86	34.16-15.71
11	64-4	10.16	11.66	6.61	21.90-10.07	25.15-11.57	14.24-6.55
12		NA	NA	NA	NA	NA	NA
13		NA	NA	NA	NA	NA	NA
14	44-5	12.87	12.87	12.41	15.57-7.16	15.57-7.16	15.01-6.91
15	50-14	5.34	5.70	5.56	30.02-13.81	13.81-32.01	31.26-14.38
16	50-14	60.70	60.70	56.03	21.99-10.12	10.12-21.99	20.30-9.34
17	50-14	7.60	9.03	6.90	24.93-11.47	11.47-29.61	22.62-10.40
18		NA	NA	NA	NA	NA	NA
19	27-0	12.32	12.32	12.54	18.25-8.40	18.25-8.40	18.57-8.54
20	27-0	13.18	14.43	16.54	22.31-10.27	24.45-11.25	28.01-12.89

Insertion time of each cluster of ERVs was estimated by two methods: (1) Phylogeny based substitution rate: LTR divergence / (distance of 3′ LTRs + dist of 5′ LTRs) [24] and (2) using the LTR divergence and fixed substitution rate of 2.3x10^-6^ and 5x10^-6^[14]. Btau, *Bos taurus*; Bind, *Bos indicus*; Bgru, *Bos grunniens*. Clusters of ERVs are showed in Figure 1. NA, the phylogenetic relationship of LTRs of clusters of ERVs did not follow the species relationship.

**Figure 3:**
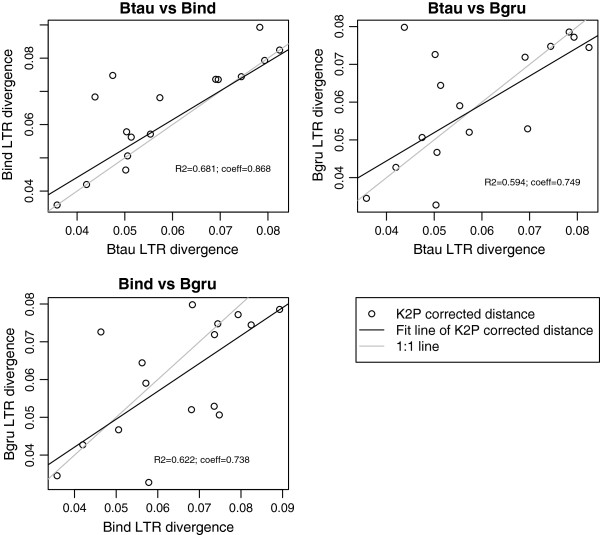
**Divergence of LTRs of clusters of ERVs from *Bos *species.** Divergence of LTRs (K2P corrected distance) of each cluster of ERV compared in pairs. Btau, *Bos taurus*; Bind, *Bos indicus*; Bgru, *Bos grunniens*.

The use of methods of LTR divergence rely on the assumption that the LTR divergences are constant between species and the violation of this assumption complicate the use of molecular clock and, therefore, the use of these methods, so the LTR divergence of each species was compared (Figure 3). In the case of ERVs from cattle and zebus, the divergence of the LTRs was similar because the regression coefficient (β) based on the Kimura 2 parameter distance was 0.868 (Figure 3). However, in both the cattle-yak and zebu-yak comparisons, the differences were higher (β = 0.749 and β = 0.738, respectively) (Figure 3).

**Figure 4:**
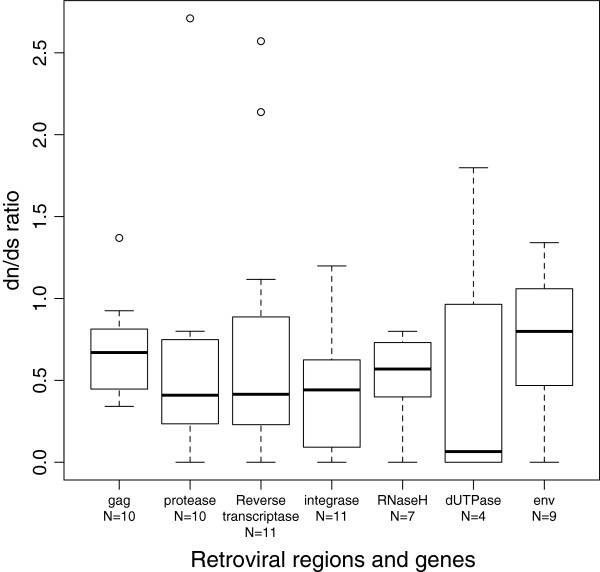
**dn/ds ratios of clusters of ERVs from *Bos *species.** Boxplot of the dn/ds ratios of retroviral genes or regions of retroviral genes of clusters of ERVs in the 3 species. N, number of clusters analyzed.

Clustered ERVs were also used to assess selective pressure. Most retroviral genes and regions of genes of clustered of ERVs were under purifying selection because their dn/ds ratio was <1 (Figure 4). However, in some clustered of ERVs, the dn/ds ratio was greater than 1, especially in the dUTPase and reverse transcriptase regions (Figure 4).

## Discussion

In this work, the variability in the number of bovine endogenous retroviruses and in the retrovirus families represented was analyzed in different species of the Bovidae family with the aim of improving our knowledge of these genomic elements in bovids.

In cattle, approximately 13,000 putative ERVs were previously detected in Btau_3.1 genome version for cattle using three different computational tools (BLAST, LTR_STRUC and RetroTector©) [4]. In this work, the Bos_3.1_UMD genome version was analysed using LTRharvest and LTRdigest. These programs were selected due to their advantages with respect to the speed of the analysis and the possibility of customization.

The results obtained in this genome version combining LTRharvest and LTRdigest suggested that 1532 elements could be endogenous retroviruses, being 574 of them detected in our previous work [4]. The detection of new putative ERVs could be a consequence of the use of a newer version of the assembly of the cattle genome and the use of different programs. In our previous works [4,13], most of the ERVs were detected by only one program while the ERVs detected by two programs or more were minority As previously suggested [25], to detect transposable elements the use of different computational tools is advisable since each tool gives different information. So, the use of another approach, in this case LTRharvest and LTR digest, followed this trend and new ERVs could be detected.

LTRharvest finds elements with LTRs, so in the case of ERVs that have lost one of their LTRs or one of the LTRs is not recognizable, they could not be detected and, therefore, analyzed. The clearest example of this limitation is the lack of detection of BoERV4, BoERV6 and BoERV14 in the analysis using LTRharvest and LTRdigest and their subsequent detection using searches based on BLAST.

### Detection of BoERVs

To the best of our knowledge, this work is the first genome-wide analysis of ERVs in zebus and yaks. Class II ERVs have previously been detected in yaks by experimental means [7]. In this work, both Class I and II ERVs were detected in both zebus and yaks.

The *de novo* search for ERVs and the specific search for BoERV families detected a total of 30 ERV families, six of which are new, in the three analyzed species of the *Bos* genus. In both analyses, more ERVs were detected in cattle than in the other two species, and zebus had an intermediate number. Two reasons could explain these differences: 1) the differences in the genome assemblies and the sequencing methods: the assembly of cattle genome has full representation of chromosomes, a coverage of 9X and sequenced using Sanger [5]; while the assembly zebu has a full representation at contig level, a coverage of 52X and sequenced using SOLiD [8]; and yak has a full representation at scaffold level, a coverage of 65X and sequenced using Illumina HiSeq and Illumina GA [9]. In addition, sometimes there are problems with the assembly of repetitive elements [25], so the lack of ERVs in zebu and yak could be an additive effect of quality of genome sequencing and the usual bias mapping repetitive elements. 2) An expansion of retroviral copies in the common ancestor of cattle and zebus and continued expansion in cattle after the split because the number of elements in cattle was twice that in the two other species.

### Evolutionary history of BoERV families

Almost all BoERV families were detected in the analyzed species of the Bovidae family. Thus, 25 of the 30 BoERV families could have been present in the common ancestor of the Bovidae family (20MYA or earlier). For these families, similar numbers of copies were detected in yaks, goats and domestic sheep, and more copies were detected in cattle and zebus. Thus, an increase in the copy number in cattle and zebus could be possible.

Three out of the four families specific to the Bovinae subfamily were from Class II, a class of ERVs related to *Betaretroviruses*. Given that 1) Class II elements are the most abundant ERVs in rats and mice [1,26], 2) an ERV family with recent activity in humans, the HERV-K family [16], belongs to Class II and 3) the most studied ERV of domestic sheep [2,27] is the endogenous counterpart of JSRV virus, a *Betaretrovirus*, Class II retroviruses could have been active until recently in some groups of mammals.

The high number of reads that mapped to the BoERV15 and BoERV29 families in water buffalo, bighorn sheep and Dall sheep is difficult to understand because the copy numbers of these families were not remarkable in *Bos* species, domestic sheep or goats. The reads in BoERV15 were located between positions 1 and 250 of the reverse transcriptase sequence used, and this segment is highly similar to the Bov-B LINE/SINE, a transposable element present in some vertebrates [28]. The high number of reads that mapped to these families could be a) due to their similarity to other genomic elements, or b) that these two families were more successful in these species.

### Clustered ERVs and evolutionary implications

The estimation of the integration time of ERVs based on LTR divergence is a controversial issue due to several limitations of this approach, such as gene conversion between LTRs [15]. This work provides a convenient scenario in which to test the usefulness of LTR divergence as a measure of the integration time. In this work, two different methods were used: one based on a phylogeny-based substitution rate and the other based on a fixed substitution rate. Discrepancies between species have arisen. Thus, the comparison of the divergences of clustered ERVs could be helpful in this scenario.

We assumed that clustered ERVs should have similar levels of divergence in their LTRs, and we found that the deviation from this assumption was lower in the cattle-zebu comparison than in the cattle-yak and zebu-yak comparisons. Thus, among other conclusions [15], it appears that divergence between species could also affect the divergence of LTRs, most likely due to the differences in the genomes since their split. In the case of yaks, genomic differences relative to cattle have been detected due to the adaptation of yaks to high altitudes [9].

The purifying selection acting on ERVs in some specific families or groups is a general phenomenon that has been detected in humans [1,16,17] and crocodiles [18], mainly in the protease, reverse transcriptase and *env* genes of ERVs. In addition, purifying selection was detected in a homologous *env* gene retrieved from 8 simian species [29]. In this work, the selection analyses were extended to *gag* gene and *pol* gene was analyzed in-depth, so in total seven genes or regions of retroviral genes of clustered of ERVs from different species were analyzed. In addition to the *env* gene, purifying selection was also detected in the *gag*, dUTPase, integrase and RnaseH genes. The evolutionary conservation of clustered ERVs detected in this work suggests the lack of detrimental effects of these proviruses on their hosts. Thus, we can speculate that the genes or regions of genes from these ERVs could be a reservoir of functional elements that could be domesticated. Similarly, purifying selection has been detected in domesticated genes such as the syncitin genes of ruminants [30], carnivore [31] and primates [29,32].

## Conclusions

In this study, previously described bovine endogenous retrovirus families were detected in other members of the Bovidae family. In addition, six previously undescribed ERV families were characterized. Among all these families, 25 were found in more than one species of the Bovidae family. Thus, most of these BoERV families could have been present in the common ancestor of the Bovidae.

Most of the specific ERV families detected in species from the Bovinae subfamily were from Class II, a retroviral class active in other mammals. In addition, the high number of BoERVs detected in cattle suggests that an additional expansion of retroviral copies could have occurred in this species.

When two methods to estimate LTR divergence as a measure of the insertion time were compared, a deviation in the LTR divergence of clusters of retroviral copies was detected, adding new concerns regarding the use of LTRs to estimate the insertion time of ERV copies, that is, the violation of the molecular clock. Purifying selection acting on clusters of ERVs from different species of the *Bos* genus was detected, extending the detection of this type of selection to new ERV regions (integrase region of *pol* gene, for instance) and detecting purifying selection among other *Bos* species.

## Methods

### Detection and characterization of endogenous retroviruses in the Bos genus

The cattle genome (*Bos taurus*, Bos_3.1_UMD version) [5] and contigs from the whole-genome sequencing projects for zebus (*Bos indicus*, Bos_indicus_1.0 version, GenBank: AGFL00000000) [8] and yaks (*Bos grunniens*, GenBank: AGSK00000000) [9] were retrieved from the NCBI server (Table 4).

**Table 4:** Features of data analyzed in this work

**Species / breed**	**Kind of data**	**Version or accession**	**Reference**	**Bases analyzed**	**Average read length**
*Bos taurus*	Assembly	Bos_3.1_UMD	[5]	2,640,420,000	-
*Bos indicus*	Contigs	GenBank:AGFL00000000	[8]	2,475,930,000	-
*Bos grunniens*	Contigs	GenBank:AGSK00000000	[9]	2,525,000,000	-
*Ovis aries*	Assembly	Oar_v3.1	[12]	2,587,520,000	-
*Bubalus bubalis*	Contigs	Genbank:ACZF000000000		603,180,000	-
*Bubalus bubalis*	Reads	SRA project number: SRP001574	[10]	54,286,293,184	101
*Ovis canadensis*	Reads	SRA run numbers: SRR501858, SRR501895 and SRR501898	-	511,945,904	202
*Ovis dalli*	Reads	SRA run numbers: SRR501847 and SRR501897	-	70,954,646,048	202
*Capra hircus*	Contigs	Genbank: AJPT00000000	[11]	2,524,660,000	-

For each species, bovine endogenous retroviruses (BoERVs) were detected using the programs LTRharvest [19] and LTRdigest [20] included in GenomeTools. In the case of LTRdigest, to search for retroviral genes, the option of using HMMER was chosen, and the Hidden Markov Model profiles of retroviral genera (*Alpharetrovirus*, *Betaretrovirus*, *Deltaretrovirus*, *Gammaretrovirus*, *Epsilonretrovirus*, *Lentivirus* and *Spumavirus*) and genes/regions (*gag*, protease, reverse transcriptase, ribonuclease H, integrase, *env*, dUTPase and ORFX) were retrieved from the Gypsy Database [33].

To find conserved reverse transcriptase sequences and to enable comparisons with our previous work [4], 1) a search for reverse transcriptase sequences was performed in the sequences detected by LTRharvest using well-annotated reverse transcriptase regions and the BLAST program [34], and only results with an identity >90% and a length of >500 bases were taken into account; and 2) the sequences detected in this search using BLAST (300 in cattle, 156 in zebus, 70 in yaks), the sequences of the 24 BoERV families [4] and the sequences of 11 exogenous retroviruses (bovine foamy virus (BFV, GenBank: NC_001831), bovine leukemia virus (BLV, GenBank: NC_001414), feline leukemia virus (FeLV, GenBank: NC_001940), human immunodeficiency virus (HIV, GenBank: NC_001802), human T-lymphotropic virus (HTLV GenBank: NC_001436), human spumaretrovirus (HSRV, GenBank: NC_001795), Jaagsiekte sheep retrovirus (JSRV, GenBank: NC_001494), Mason-Pfizer monkey virus (MPMV, GenBank: NC_001550), murine leukemia virus (MLV, GenBank: NC_001501), Visna virus (GenBank: NC_001452) and the ZAM element (GenBank: AJ000387)) were aligned using the E-INS-i strategy implemented in MAFFT 6.833b [35]. A phylogenetic analysis was performed with these sequences using the neighbor-joining method implemented in MEGA5 [36]. The following options were used: p-distance, pairwise deletion and 1000 bootstrap replicates. In addition, Bayesian inference was used to construct a phylogenetic tree. The phylogenetic analysis have been deposited in TreeBASE with the accession S14634. MrBayes 3.2 [37] was used with the options lset = 6, rates = invgamma and 10^6^ generations; the 25% of first trees were discarded. Because the Bayesian tree was similar to the tree generated with the neighbor-joining method, the Bayesian tree is not shown.

### Detection of BoERVs in other ruminant species

The genome of domestic sheep (*Ovis aries*, Oar_v3.1) [12] and contigs from the whole-genome sequencing projects for water buffalo (*Bubalus bubalis*, Bubalus_bubalis_Jaffrabadi_v2.0, GenBank: ACZF000000000) and goats (*Capra hircus*, GenBank: AJPT00000000) [11] were retrieved from the NCBI server (Table 4).

For each species, a BLAST search for each of the reverse transcriptase genes of the 24 BoERV families and the six new families detected in this work was performed, and only results with a length of >500 bases and an e-value <10^-15^ were taken into account. To avoid detecting the same ERV copy in different contigs or assembly errors, CD-HIT [38] was used to detect identical sequences (options: -c 1, -n 8). After removing the identical sequences, the detected sequences and the 30 BoERV families were aligned using the E-INS-i strategy implemented in MAFFT 6.833b, and a phylogenetic analysis was performed using the neighbor-joining method implemented in MEGA5 (options used: p-distance, pairwise deletion and 100 bootstrap replicates). Based on these analyses, the number of copies of each family was estimated.

To compare the results with the species of the *Bos* genus, the same procedure was used in cattle, zebus and yaks.

Because the results for water buffalo were not revealing due to the low coverage, data available for the Murrah breed [10] were analyzed (Table 4). The reads (SRA project number: SRP001574) were mapped against the reverse transcriptase sequences of the 30 BoERV families using the NUCmer program in MUMmer 3.1 [39]. Only reads with >95% identity were considered. Reads for bighorn sheep (*Ovis canadensis*, SRA run numbers: SRR501858, SRR501895 and SRR501898) and Dall sheep (*Ovis dalli*, SRA run numbers: SRR501847 and SRR501897) were analyzed using the same procedure (Table 4).

### Evolutionary analyses of clusters of BoERVs

The phylogenetic analysis revealed 20 cases in which ERVs from the three species were significantly clustered altogether. Two evolutionary analyses were performed for these clusters of sequences:

a) Based on LTR sequences. The integration time was calculated by two methods: (1) applying an estimated substitution rate as proposed in [24] and (2) applying a fixed substitution rate [14] to the LTR divergence. For each cluster of ERV, the sequences annotated as LTRs by LTRharvest were retrieved. To estimate the substitution rate, the six LTRs of a trio were aligned using the L-INS-i strategy implemented in MAFFT 6.833b. A maximum-likelihood phylogeny was constructed using PhyML [40] (GTR + G + I model, optimizing the α parameter and invariable sites), and the distances between the 5′ and 3′ LTRs of yaks and cattle/zebus were retrieved and applied as described in [24] to estimate a substitution ratio. To estimate the LTR divergence, the pairs of LTRs for each cluster of ERV were aligned using the L-INS-i strategy implemented in MAFFT 6.833b, and the number of differences was counted. Then, an estimated divergence rate and a fixed divergence rate (2.3x10^-6^ - 5x10^-6^ substitutions/site/year) were applied to estimate the integration time of each “trio.” In addition, the divergences of closely related BoERVs were compared (cattle vs. zebu, cattle vs. yak and zebu vs. yak). In each comparison, a linear regression was performed using R [41].

b) Based on retroviral genes. The selective pressure affecting retroviral genes was analyzed. The different genes or regions of retroviral genes detected by LTRdigest for each trio were aligned using the E-INS-i strategy implemented in MAFFT 6.833b. Selection analysis was performed using PAML4.4 [42] using the models M0, M7 and M8 and choosing the best model using a likelihood-ratio test. The most extreme values (dn = 0 or ds = 0) were discarded.

## Competing interests

The authors declare that they have no competing interests.

## Authors’ contributions

BMJ conceived the study; KGE & BMJ designed the study; KGE performed the analyses. Both authors analyzed the results and wrote the paper. Both authors read and approved the final manuscript.

## Supplementary Material

Additional file 1 **Chromosomal position of detected endogenous retroviruses in *Bos taurus *** **in simple BED format.** Click here for file

Additional file 2 **GI number and position of detected endogenous retroviruses in *Bos indicus *** **in simple BED format.** Click here for file

Additional file 3 **GI number and position of detected endogenous retroviruses in *Bos grunniens *** **in simple BED format.** Click here for file
